# The enhancement effect of estradiol on contextual fear conditioning in female mice

**DOI:** 10.1371/journal.pone.0197441

**Published:** 2018-05-15

**Authors:** Yui K. Matsumoto, Masanori Kasai, Kazuya Tomihara

**Affiliations:** 1 Department of Chemistry and Bioscience, Faculty of Science, Kagoshima University, Kagoshima, Japan; 2 Department of Chemistry and Bioscience, Faculty of Science, Graduate School of Science and Engineering, Kagoshima University, Kagoshima, Japan; 3 Department of Psychology, Faculty of Law, Economics and Humanities, Kagoshima University, Kagoshima, Japan; Radboud University Medical Centre, NETHERLANDS

## Abstract

Several studies have reported regulatory effects of estrogens on fear conditioning in female rodents. However, these studies used different doses, durations, and/or administration methods, and reported inconsistent results. To clarify the effect of estrogen on fear conditioning, we investigated the effects of different doses and durations of estradiol administration on freezing behavior during contextual fear conditioning in ovariectomized (OVX) mice. In Experiment 1, OVX ICR mice received a single subcutaneous (s.c.) injection of either oil vehicle (control, 0.1 ml sesame oil) or varied doses (0.5 μg/0.1 ml, 5 μg/0.1 ml, or 50 μg/0.1 ml) of 17β-estradiol-3-benzoate (EB). Fear conditioning was conducted two days post-EB treatment, and the mice were tested for the learned fear response the following day. In Experiment 2, OVX female mice received an s.c. implantation of a Silastic capsule (I.D. 1.98 × 20.0 mm) containing either vehicle or varied doses (0.05 μg/0.1 ml, 0.5 μg/0.1 ml, 5 μg/0.1 ml, 50 μg/0.1 ml) of EB. Two weeks after implantation, fear conditioning was conducted. During the tests conducted 24 h after conditioning, the high dose EB group showed longer freezing times in both experiments, and lower locomotor activity compared to the control or lower dose groups. In Experiment 3, serum estradiol concentrations of the mice that were treated like those in Experiment 2, were measured; the serum levels of estradiol increased linearly according to the dose of EB administered. The results suggest that mice treated with a high dose of EB exhibit enhanced fear learning, regardless of treatment duration. As a woman’s vulnerability to emotional disorders increases in the peripregnancy period, during which estrogen levels are high, the results from the high-dose EB groups may be important for understanding the hormonal mechanisms involved in these disorders.

## Introduction

Changes in steroid hormone levels during premenstrual, postpartum, and postmenopausal periods can lead to several disorders associated with mood, cognition, emotion, learning, and memory [1–4]. Estrogens are hormones that are intimately involved in the regulation of these disorders. It has been reported that memory and cognitive performance can change with fluctuations in circulating estradiol levels throughout menstruation, pregnancy, or the menopausal period [5–7]. Additionally, estrogen replacement therapy has been found to alleviate memory impairments in postmenopausal and surgically menopausal women [8, 9], and animal studies have shown that estradiol administration increases performance in a spatial memory test in ovariectomized (OVX) rats [10, 11]. Thus, estrogen is considered to play an important role in learning and memory regulation.

However, the effects of estrogen on learning and memory, especially emotional learning such as fear conditioning, are still controversial. Studies have reported that estrogen administration to OVX animals decreases the freezing response in fear conditioning tests [12–15]. However, other studies have reported that estrogen administration increases conditioned fear [15, 16]. The inconsistency in these results may, at least partially, be due to methodological variations between studies. First, the variation in the doses used between the studies could have a critical effect on behavior in fear conditioning tests. For instance, Barha *et al*. [15] found that low-dose estrogen administration facilitated, whereas high- and mid-dose estrogen administration impaired, contextual fear conditioning in female OVX rats. Similar dose-dependent variations in estrogenic effects were also shown in anxiety-related behavioral tests in mice [17, 18], and non-spatial working memory tests in rats [19]. In humans, it has been reported that cognitive performance in working memory tasks with emotional stimuli can vary with fluctuations in circulating hormonal levels throughout the menstrual period [7]. Thus, the effects of various estradiol levels seem likely to share common behavioral mechanisms. Second, variations between studies in terms of estradiol administration can affect the behavior of mice in fear conditioning tests. However, no study has investigated the effects of differences in administration duration on fear conditioning. One study that examined the acute effects of a single estradiol injection reported both inhibition and promotion of fear memory [15]; however, it is unclear whether estradiol injections have an inhibitory or stimulatory effect on fear memory with long-term treatment. Additionally, the effects of administration duration could interact with the effects of the doses of estradiol described above.

We hypothesized that chronic treatment with high doses of estradiol unlike acute treatment would facilitate fear conditioning in female mice, while low doses of the hormone would inhibit fear. In fact, similar effects of chronic treatment on anxiety-related behaviors were found in our previous studies [17, 18]. To test this hypothesis, we investigated fear conditioning in OVX mice that were treated acutely (Experiment 1) and chronically (Experiment 2) with varying doses of estradiol.

## Methods

### Ethics

All experimental procedures were conducted in strict accordance with the Guidelines on the Care and Use of Laboratory Animals in Kagoshima University, Japan, and approved by the Ethics Committee for Animal Experimentation at Kagoshima University (#LE10007).

### Animals

Eight-week-old female ICR mice were obtained from Kyudo Co. (experimental animals in Experiment 1: N = 32, Experiment 2: N = 40, Experiment 3: N = 53; Kumamoto, Japan). They were housed in groups (4–5 per cage) in Plexiglas cages (30 × 20 × 12 cm) until surgery. After surgery, they were individually housed in smaller Plexiglas cages (10.5 × 17.5 × 11 cm). In the present study, abnormal behaviors induced by isolation, such as hyperactivity and depressive-like behaviors, were not found during daily observations. We performed surgery, hormonal treatment, fear conditioning tests, and measurements of uterine weight, and collected blood for hormonal assays when all the mice were 10–12 weeks old. Throughout the experimental period, the mice were kept in a controlled environment at 23 ± 2°C, with a semi-reversed 12/12 h light/dark cycle (lights off at 12:00). Food and water were available *ad libitum*.

### Hormone treatments

**Fig 1:**
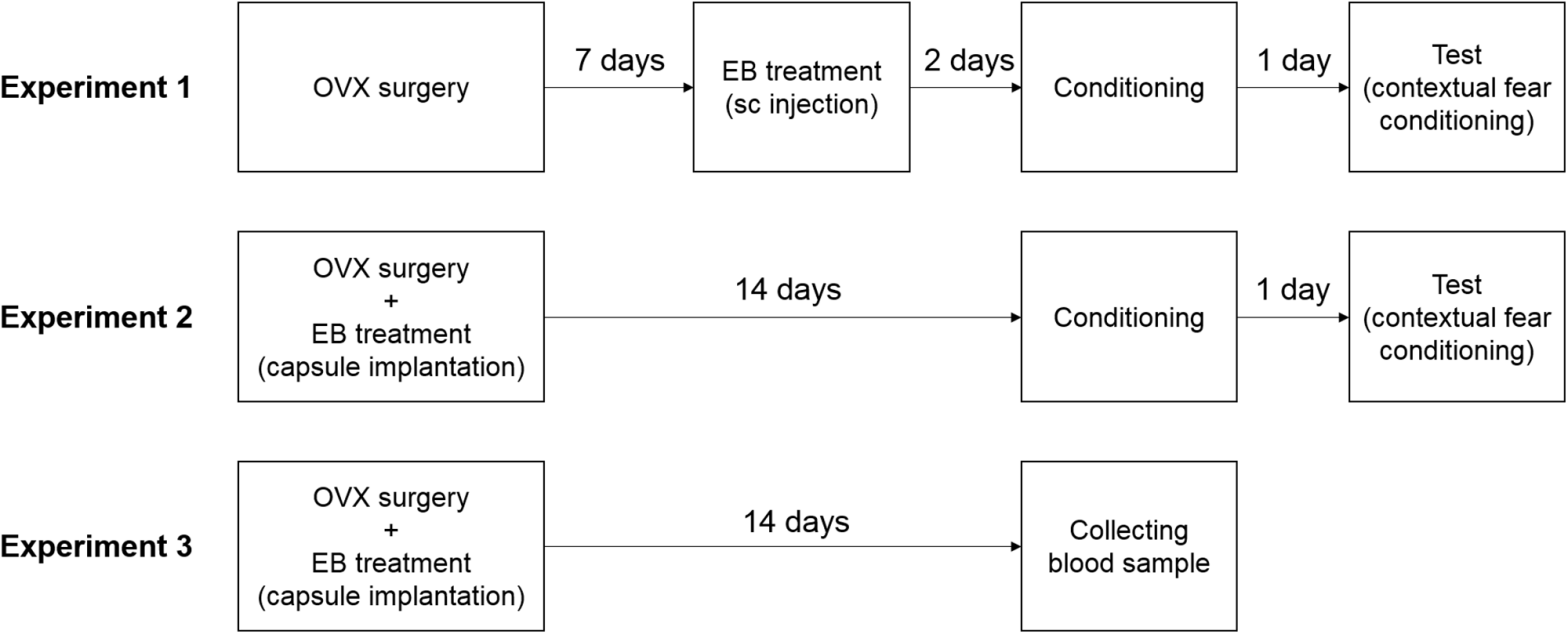
Experimental procedure. In Experiment 1, mice received a single s.c. injection of either oil vehicle (control, 0.1 ml sesame oil) or various doses (0.5 μg/0.1 ml, 5 μg/0.1 ml, or 50 μg/0.1 ml) of EB 7 days post-ovariectomy. Fear conditioning was conducted 2 days after EB treatment, and the mice were tested for their conditioned fear responses the following day. In Experiment 2, mice were ovariectomized and implanted s.c. with a Silastic capsule (I.D. 1.98 × 20.0 mm) containing either vehicle or various doses (0.05 μg, 0.5 μg, 5 μg, 50 μg/0.1 ml) of EB. Two weeks post-implantation, fear conditioning and testing were conducted. In the conditioning phase, 3 min after being placed in the chamber, mice were administered three consecutive foot shocks (duration: 2 s, 0.8 mA) with 30 s intershock intervals. The day after the last behavioral test, the animals were sacrificed using a pentobarbital overdose. The uteri were collected and the wet weights were recorded. In Experiment 3, mice were ovariectomized and implanted (s.c.) with a Silastic capsule (I.D. 1.98 × 20.0 mm) containing either vehicle or various doses (0.05 μg, 0.5 μg, 5 μg, 50 μg/0.1 ml) of EB. Two weeks post-implantation, the animals were decapitated and their trunk blood was collected for the hormonal assay.

A graphical representation of the experimental procedure in this study is shown in Fig 1. In Experiment 1, all mice were bilaterally ovariectomized under pentobarbital anesthesia (80 mg/kg) approximately one week after arrival. One week post-surgery, mice were randomly assigned to four 17β-estradiol-3-benzoate (EB) treatment groups, and the animals in each group received a single subcutaneous (s.c.) injection of 0.1 ml of either 0.5 μg (EB0.5S, n = 9), 5 μg (EB5S, n = 7), or 50 μg (EB50S, n = 8) EB in oil, or oil vehicle alone (EB0S, n = 8). All injections were administered between 7:00 and 10:00 a.m., and conditioning trials were conducted approximately 48 h after injections.

In Experiment 2, the mice were bilaterally ovariectomized approximately one week after arrival. At the time of surgery, the mice were randomly assigned to five treatment groups and implanted s.c. with a Silastic capsule (I.D. 1.98 mm, O.D. 3.18 mm × 20.0 mm; Silastic Laboratory Tubing, Dow Corning CO, Midland, MI, USA) containing either an oil vehicle (EB0L, n = 8) or various EB concentrations: 0.05 μg/0.1 ml (EB0.05L, n = 8), 0.5 μg/0.1 ml (EB0.5L, n = 8), 5 μg/0.1 ml (EB5L, n = 8), or 50 μg/0.1 ml (EB50L, n = 8). Since the capsules contained approximately 0.06 ml of liquid, the actual EB dose inside the capsules was approximately 0.03, 0.3, 3, or 30 μg, respectively. Conditioning trials were conducted 14 days post-surgical implantation. Our groups were based on the EB doses used in a previous study [17] that revealed an effect of estrogen dose on anxiety-like behavior in order to allow for a comparison to be made.

In Experiment 3, animals that received hormone treatments identical to that used for animals in Experiment 2 were used for the estradiol assay (EB0L, n = 8; EB0.05L, n = 7; EB0.5L, n = 8; EB5L, n = 4; EB50L, n = 8). Two weeks after capsule implantation, the animals were decapitated and their trunk blood was collected. The collected samples were then left for 1 h at room temperature before centrifugation for 15 minutes at 3000 × g at 4°C. The supernatant was collected and stored at −80°C until the assay was performed. Serum estradiol concentrations were determined using a chemiluminescent immunoassay (CLIA), which was performed by Clinical Pathology Laboratory Inc. Co. Ltd. (detection threshold: 12 pg/ml; Kagoshima, Japan). Serum estradiol levels in almost all animals in the EB0L, EB0.05L, and EB0.5L groups were undetectable, because they were below the detection threshold. Therefore, additional animals were used for the estradiol assay for these groups (EB0L, n = 4; EB0.05L, n = 7; EB0.5L, n = 7), and the serum estradiol concentrations were subsequently determined using liquid chromatography-tandem mass spectrometry (LC-MS/MS) by ASKA Pharmaceutical Medical Inc. Co. Ltd. (detection threshold: 0.5 pg/assay; Kawasaki, Japan), which has higher sensitivity and accuracy than the CLIA.

### Behavioral testing

Transparent plastic chambers (30 × 10 × 19 cm, height 15 cm; O’Hara & Co., Ltd, Tokyo, Japan) were used for the conditioning and test trials. The floor consisted of 19 stainless steel rods connected to a shock generator.

The contextual fear conditioning procedure was based on an established paradigm [20]. The experiment was conducted over two consecutive days during the light cycle, and involved a conditioning and testing day. On the first day, the mice were transported to the experimental room 1 h before conditioning, and subsequently placed in the chamber. Three minutes after being placed in the chamber, the mice were administered three consecutive foot shocks (duration: 2 s, 0.8 mA) with 30 s intershock intervals. Thirty seconds after the final shock, the mice were returned to their home cages and then returned to the colony room. The next day, approximately 24 h after conditioning, the mice were transported back to the experimental room in the same way as on the conditioning day.

To assess conditioned fear, the mice were placed in the chamber again. During the 10-min test, all behaviors exhibited in the chamber were recorded using a video camera (Sony Handycam, DCR-HC62, Tokyo, Japan) positioned in front of the chamber. After testing, the cumulative duration of specific behaviors was determined from the recorded video by a well-trained observer blinded to the experimental hypothesis. The behaviors scored were freezing (absence of all movements except those associated with respiration), locomotion (walking and running around the chamber), stretching (stretching the whole body forward while keeping the hind limbs in place), tail writhing (sinuous movement of the tail, with or without banging on the floor), and grooming (licking and washing of the face and body). The total duration of freezing, which is a fear-related behavior, was defined as a measure of fear-related memory [21, 22]. The total duration of locomotion and rearing served as a measure of activity and exploratory behavior. Stretching, tail writhing, and grooming indicated a state of tension [21, 22]. The day after the last behavioral test, the animals were sacrificed using a pentobarbital overdose. The uteri were collected and the wet weights were recorded.

### Statistical analysis

The total time of each behavior, uterine weight, and serum concentrations of estradiol were analyzed by a one-way analysis of variance (ANOVA) followed by a Tukey’s test using Prism software (GraphPad Prism, San Diego, USA). The serum estradiol values were log-transformed (Log_10_) before the ANOVA was performed to maintain the homogeneity of variance. To estimate the differences in the estradiol levels between the groups, we compared the levels that had been measured by the two different assay methods for the five treatment groups. To further investigate the effects of estrogens on fear learning, we used linear and quadratic regression analyses using SPSS v.20 (IBM Corp., Armonk, NY) to assess the association between the cumulative duration of freezing and the uterine weights in Experiment 2, which were expected to be an index of circulating estrogen levels. All data are presented as means ± standard error of the mean (SEM). The data for one mouse, which was injured during conditioning, were excluded from the statistical analyses.

## Results

### Enhancement effects of estradiol benzoate on contextual freezing behavior

**Fig 2:**
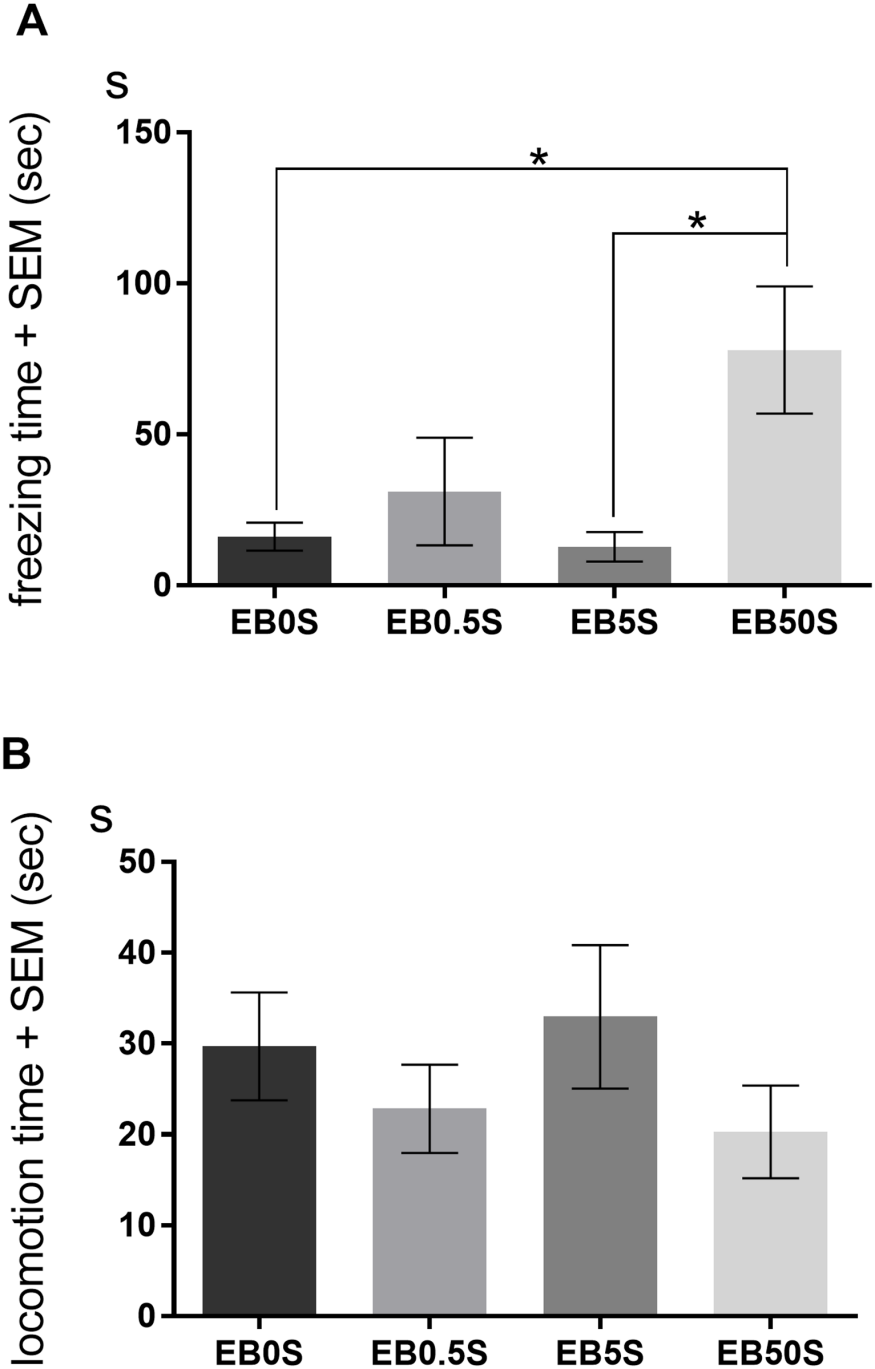
Effect of freezing and locomotion durations on contextual fear conditioning in Experiment 1. OVX mice received a single s.c. injection of EB at a dose of 0.5 μg/0.1 ml (EB0.5S), 5 μg/0.1 ml (EB5S), or 50 μg/0.1 ml (EB50S), or an oil vehicle (EB0S) two days before conditioning. The mean (± SEM) duration of freezing (A) and locomotion (B) in the 10-min test conducted 24 h after conditioning is shown. Mice treated with a high dose of EB (EB50S) displayed significantly more freezing than control and EB5S mice (*p* < 0.05). Significant differences are denoted by an asterisk; *p < 0.05.

In Experiment 1, we examined the effect of a single EB injection on conditioned fear memory acquisition. In OVX female mice treated with a high-dose of EB, we found an increase in fear memory. Specifically, there was a significant main effect of EB dose on total freezing time (F(3, 28) = 5.00, *p* < 0.05). Post hoc comparisons showed that the mice treated with EB50S spent more time freezing than controls (EB0S; *p* < 0.05) and mice treated with EB5S (*p* < 0.05), but not those treated with EB0.5S (Fig 2A). We did not find a significant difference in locomotion time among the groups (Fig 2B). There were also no significant main effects of EB treatment on other behaviors (S1 Table).

**Fig 3:**
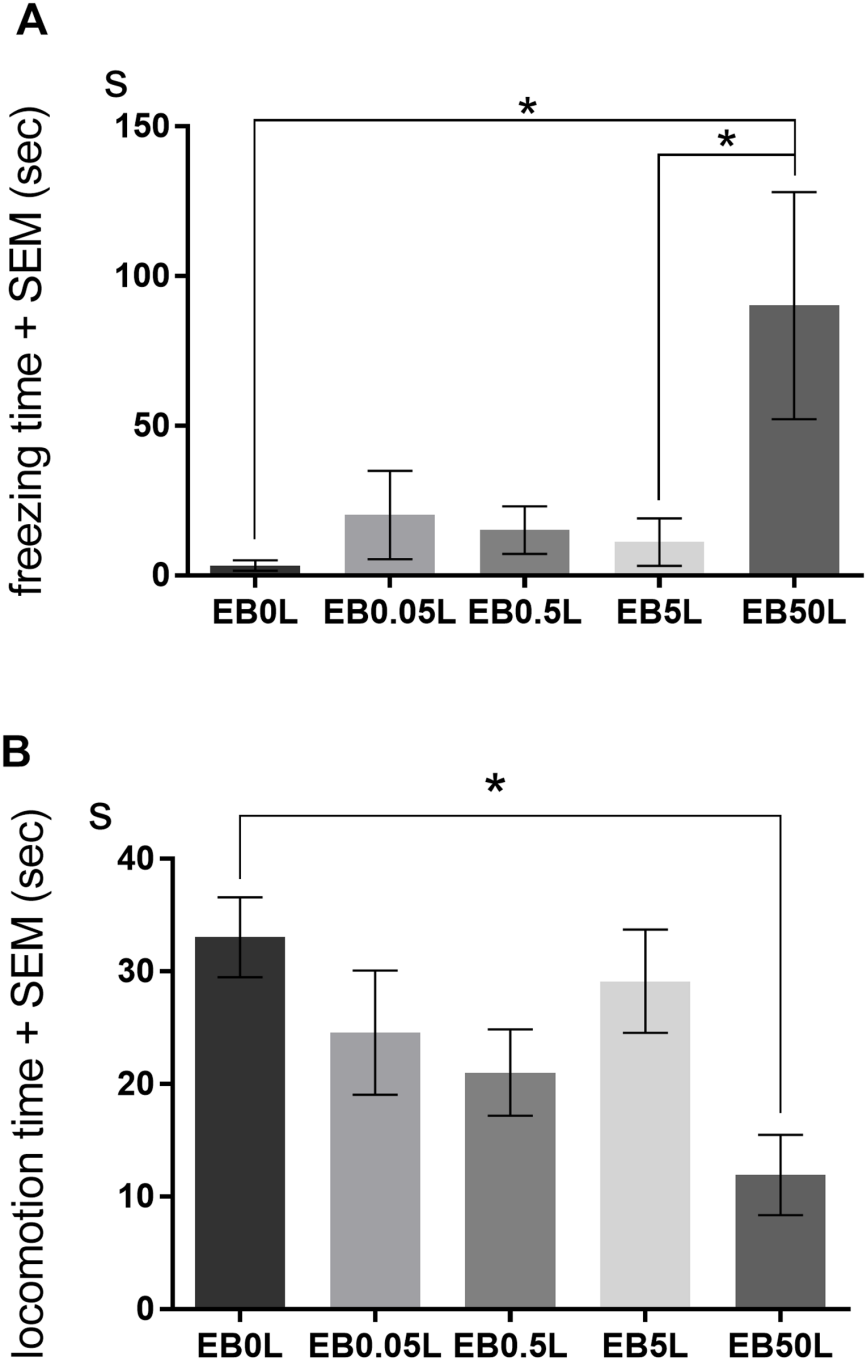
Effect of freezing and locomotion durations on contextual fear conditioning in Experiment 2. OVX mice were implanted s.c. with a Silastic capsule containing either vehicle (EB0L), 0.05 μg/0.1 ml (EB0.05L), 0.5 μg/0.1 ml (EB0.5L), 5 μg/0.1 ml (EB5L), or 50 μg/0.1 ml (EB50L) 14 days before conditioning. Mean (± SEM) duration of freezing (A) and locomotion (B) in the 10 min test conducted 24 h after conditioning. Mice treated with EB50L showed a significantly longer freezing time compared with control (*p* < 0.05) and EB5L (*p* < 0.05) mice. EB50L mice also displayed a significantly shorter locomotion time compared to control mice (*p* < 0.05). Significant differences are denoted by an asterisk; *p < 0.05.

In Experiment 2, we examined the effect of chronic EB treatment on fear conditioning in female mice. We found that chronic treatment with a high dose of EB stimulated fear learning, with a significant main effect of dose on total freezing time (F(4, 35) = 3.49, *p* < 0.05). Post hoc comparisons showed that EB50L mice displayed more freezing than control (EB0L) and EB5L mice (*ps* < 0.05; Fig 3A). Similarly, there was a significant main effect of EB treatment on locomotion time (F(4, 35) = 3.59, *p* < 0.05), with EB50L mice showing significantly less locomotion than EB0L mice (*p* < 0.05; Fig 3B). In addition, there was also a marginal difference between the EB50L and EB5L mice with regard to locomotion time (*p* = 0.054; Fig 3B). EB5L mice displayed more tail tremors than control mice (19.0 ± 5.4 versus 3.4 ± 1.2) (F(4, 35) = 2.987, *p* < 0.05; S2 Table). There was no significant effect of EB treatment on the duration of other behaviors, although we found a slight increase in stretching time in the higher-dose groups compared with the controls (S2 Table).

### Hormonal effects

An s.c. injection of EB is often used to induce a state of estrus in females, and uterine weights gradually increase with increasing doses of EB [23, 24]. To examine how the injection of EB affects circulating estrogen levels, we determined the uterine weights of the animals after behavioral testing. In Experiment 1, the uterine weights increased with increases in EB dose, although they were measured 5 days after EB administration (Table 1: EB0S, n = 7; EB0.5S, n = 6; EB5S, n = 7; EB50S, n = 7; F(3, 23) = 5.52, *p* < 0.01). The uterine weight in the EB50S (52.9 ± 6.2 mg) group was significantly higher than that in the EB0S (29.6 ± 2.0 mg) group (*p* < 0.01). We also measured the uterine weight after behavioral testing in Experiment 2, and the weight was found to increase with increasing EB dose (Table 1: EB0L, n = 6; EB0.05L, n = 6; EB0.5L, n = 7; EB5L, n = 7; EB50L, n = 7; F(4, 28) = 34.60, *p* < 0.0001). Uterine weights in the EB5L (173.6 ± 24.8 mg) and EB50L (193.7 ± 13.9 mg) groups were significantly higher than those of the vehicle (27.8 ± 1.4 mg), EB0.05L (25.3 ± 1.2 mg), and EB0.5L (51.9 ± 7.7 mg) groups (*p <* 0.0001). Thus, the uterine weights were affected by the EB dose used.

**Table 1:** Uterine weight.

**Experiment 1**					
EB dose	EB0S	EB0.5S	EB5S	EB50S	
Uterine weight (mg)	29.6 ± 2.0^a^	36.8 ± 2.6	41.9 ± 4.4	52.9 ± 6.2^b^	
**Experiment 2**					
EB dose	EB0L	EB0.05L	EB0.5L	EB5L	EB50L
Uterine weight (mg)	27.8 ± 1.4^a^	25.3 ± 1.2^a^	51.9 ± 7.7^a^	173.6 ± 24.8^b^	193.7 ± 13.9^b^

Uterine weights after the test in Experiment 1 and Experiment 2. The weights were measured 5 days after a single administration of EB in Experiment 1, and 17 days after implantation of EB capsules in Experiment 2. The uterine weights increased in line with the EB dose administered. The superscript letters indicate statistical significance; *ps* < 0.01.

In addition, serum estradiol concentrations were measured in Experiment 3 2 weeks post-capsule implantation to examine the relationship between EB dose and serum estradiol levels. The serum levels of estradiol increased according to the dose of EB used (F(4, 25) = 55.30, *p* < 0.0001). The estradiol levels were significantly different between the groups (EB0.5L: 4.90 ± 1.11 pg/ml; EB5L: 17.75 ± 1.70 pg/ml; EB50L: 88.13 ± 14.30 pg/ml; *ps* < 0.05) but not between the EB0L (0.59 ± 0.18 pg/ml) and EB0.05L (1.95 ± 0.72 pg/ml) groups (*p* = 0.22). Thus, exogenous EB affects serum estradiol concentrations depending on the dose administered. In addition, serum estradiol levels highly correlated with uterine weight in animals with assayed hormonal levels (r = 0.77, *p* < 0.01). This suggests that uterine weight is related to serum estradiol levels in animals implanted with an estrogen capsule.

### Regression analyses

We conducted both linear and quadratic regression analyses to assess the association between the cumulative freezing duration and the uterine weights in Experiment 2. The results of the regression analysis indicated that the relationship might be quadratic rather than linear (S1 Fig). The linear model did not reach significance (n = 34, R2 = 0.104, F(1,32) = 3.73, p = 0.062), while the non-linear, quadratic function model showed significance (n = 34, R2 = 0.213, F(2,31) = 4.19, p < 0.05). Even though only 21.3% of the variance was determined, a significant U-shaped quadratic relationship between the cumulative freezing duration and the uterine weights was obtained.

## Discussion

OVX mice treated both acutely and chronically with the highest dose of EB showed increased freezing behavior in the fear-conditioning test compared to the mice treated with lower EB doses or vehicle. In addition, mice treated chronically with the highest dose of EB showed a reduced locomotion time compared to the oil-control animals. These results indicate that treatment with high EB doses, both acutely and chronically, facilitates fear learning in OVX mice. In the same way, the uterine weights and the serum estradiol concentrations increased with increasing EB doses. These findings are partially consistent with our hypothesis that chronic treatment with high-dose estradiol facilitates fear conditioning in female mice. However, the results in acute treatment are inconsistent with our prediction that acute treatment with high-dose estradiol does not facilitate fear conditioning.

The serum levels of estradiol in the EB0.5L (4.90 ± 1.11 pg/ml) and EB5L (17.75 ± 1.70 pg/ml) group were within those reported for normal cycling mice in previous studies [25–28] (approximately 5–60 pg/ml), while those in the EB50L group in Experiment 3 (88.13 ± 14.30 pg/ml) were higher than those reported for the proestrus period [25–28] and were within the estradiol level in pregnant animals [29, 30] (approximately 60–150 pg/ml). As we did not actually measure the estradiol levels of animals in Experiment 1 and 2, we can only speculate about the relationship between the estradiol level and fear learning. However, uterine weights are possible alternative parameters for circulating estradiol levels, because some studies indicated that uterine weights linearly increase with increasing doses of EB [23, 24] and a significant correlation (r = 0.77, *p* < 0.01) between uterine weights and doses of EB was obtained in this study. Additionally, the uterine weights in the animals in higher-doses groups in this study were similar to those found in pseudopregnant mice [31], rather than estrous. In human, gestational estrogen levels are also consistently higher than during the estrus cycle [32], and a woman’s vulnerability to emotional disorders increases during the peripregnancy period [33]. Therefore, our results suggest that higher estradiol levels, such as those observed during the gestational period, can promote enhanced fear learning.

Usually, a single administration of approximately 5–10 μg EB followed by an injection of progesterone is used for induction of behavioral estrus of female mice [34–36]. Then, we used the highest dose of estradiol (50 μg/0.1 ml) in order to obtain “higher” estradiol levels than those during proestrus. Therefore, the facilitation on fear conditioning by the highest-dose estradiol found in Experiment1 might be pharmacological, rather than physiological. This is supported by the results of the uterine weights obtained in Experiment 1 as indicated by the significant increase in uterine weight in the EB50S group. In the preliminary experiment, we measured the uterine weight 2 days after EB injection and also found a significant increase in uterine weight with increasing EB dose (S3 Table), suggesting that estradiol levels in the EB50S group were high during fear conditioning. However, in Experiment 1 the injection procedure was used, which differed from Experiment 2 used silastic capsule procedure, and the estradiol levels during training were possibly different from memory formation, as well as testing. Hence, no conclusive evidence can be obtained for the relationship between hormonal levels and fear learning in Experiment 1. In further studies, the actual estradiol levels need to be determined for each memory stage and the influences on fear learning should be assessed.

Previous studies have reported that treatment with estradiol has both faciliatory and inhibitory effects on conditioned fear learning in rats [15]. Inconsistent with our hypothesis, our results indicate a faciliatory but not an inhibitory effect on fear learning. There are several reasons why no inhibitory effect was found in this study. First, the control mice showed less freezing time compared to those in a previous study [16]; this may be due to the lower electric current or shorter shock duration used in the fear conditioning phase. Second, it is possible that small differences, such as the effect of estradiol on the inhibition of fear learning, were not detected owing to the small number of animals used and the low statistical power of the study. Therefore, the effects of estradiol need to be examined using other shock parameters, as well as with a larger number of animals to provide greater statistical power. Third, the timing of estrogen administration also requires further examination. In Experiment 1, the conditioning trials were conducted 48 h after EB injection, while Barha *et al*., who showed bidirectional results on fear learning in rats [15], conducted trials 30 min after injection. It is well known that estrogens mediate their effects through both genomic and non-genomic pathways. Non-genomic actions, which are independent of gene transcription and protein synthesis, occur rapidly and last for 30–45 min [37]. Rapid effects of estrogens can change neural activity in hippocampus related to learning and memory [38, 39], and activation by an agonist of the G-protein coupled receptor 30 (GPR30), a putative membrane estrogen receptor, decreases anxiety within 30 min of administration [40]. Therefore, the inhibitory effect may be mainly mediated through the non-genomic activity of EB, and thus we may have failed to detect the effect for this reason. Future studies should aim to examine the effects of pathway and receptor differences on fear learning. The inhibitory effect should be confirmed in future studies examining the role of estradiol using various methods.

One important limitation of present study is that the mechanisms that facilitate the fear learning in the animals treated with high-dose EB cannot be determined. Various mechanisms can contribute to facilitation of fear leaning, such as attention, emotion, and motivation, and fear memory itself, and estrogens can affect all of them. Because we did not use a standardized method for assessing the influence of EB on memory consolidation, or an injection of EB immediately after training, we cannot establish the actual influence of EB treatment on fear memory. However, mechanism involving emotion is thought to be important for the facilitation of fear leaning in this study, rather than fear memory itself. Many animal studies have reported the effects of EB on emotional state and stress response. Several studies using chronic treatment with high-dose EB reported increased anxiety-related behaviors in female mice [18, 41]. Chronic EB treatment also increased the corticosterone level [42], and the stimulation of glucocorticoid receptor resulted in higher anxiety-like behavior [43]. A gradual increase in anxiety in mice treated with 50 μg/0.1 ml of EB has also been reported. The same procedure was used in our study and consistent results were obtained as indicated by an increase in factorial scores for anxiety-related behaviors measured using behavioral parameters from the open field, elevated plus maze, and light-dark transition tests [17]. In Experiment 2 in our study, animals in the EB50L group showed marginal suppression of locomotor activity in the fear-conditioning chamber. Overall, these findings suggest that the facilitation of fear learning in the present study is related to increased anxiety owing to EB

Although the mechanisms in the brain were not assessed in the present study, the possible mechanisms are worth mentioning. Several areas in the brain, the most crucial being the amygdala, hypothalamus, and hippocampus, may be influenced by estrogen (Reviewed in Walf and Frye, 2006; Handa, et al, 2012) [44, 45]. Especially, the hypothalamus may be strongly associated with the emotional effects of treatment with high-dose EB. It was reported that chronic treatment with estradiol impairs the glucocorticoid-dependent negative feedback of the hypothalamic–pituitary–adrenal (HPA) axis via estrogen receptors within the hypothalamus [42]. It is possible to facilitate fear conditioning by mediating hyper-activation of the HPA axis.

Another limitation of present study is that the possibility of social isolation effects cannot be ruled out, although compared to male mice, adult female mice are insusceptible to social isolation [46] and we did not find any abnormal behavior induced by isolation in this study. Despite these limitations, it is worth highlighting that our findings suggest that higher level of estrogen, comparable to that during the peripregnancy period, may induce enhanced fear learning, because that has important implications for neuroendocrinological mechanisms underlying a woman’s vulnerability to emotional disorders.

## Conclusions

In this study, we investigated fear conditioning in OVX mice that were treated acutely and chronically with various estradiol doses. Our results indicated that mice treated with a high dose of EB exhibited enhanced freezing behavior during the contextual fear conditioning test, regardless of the treatment duration. This indicates that the estrogenic modulation of fear learning is dependent on the dose of EB administered. Although this study has some limitations, our findings will contribute significantly to future studies that aim to investigate the effect of estradiol treatment on fear memory and examine the neuroendocrinological mechanisms underlying the vulnerability of women to emotional disorders.

## Supporting information

S1 Table Behaviors recorded during the conditioning test in Experiment 1. Animals received a single subcutaneous (s.c.) injection of 0.1 ml of either 0.5 μg (EB0.5S, n = 9), 5 μg (EB5S, n = 7), or 50 μg (EB50S, n = 8) EB in oil, or oil vehicle alone (EB0S, n = 8).
(DOCX)

S2 Table Behaviors recorded during the conditioning test in Experiment 2. Animals implanted s.c. with a Silastic capsule containing either 0.05 μg/0.1 ml (EB0.05L, n = 8), 0.5 μg/0.1 ml (EB0.5L, n = 8), 5 μg/0.1 ml (EB5L, n = 8), or 50 μg/0.1 ml (EB50L, n = 8) or an oil vehicle (EB0L, n = 8). Superscript letters indicate statistical significance; p<0.05.
(DOCX)

S3 Table Uterine weight 2 days after s.c. administration of EB. Uterine weights were measured 2 days after a single administration of EB in a second group of animals. Animals received an s.c. injection of either oil vehicle (control, 0.1 ml sesame oil (EB0S), n = 8) or various doses (1 μg/0.1 ml (EB1S), n = 8; 5 μg/0.1 ml (EB5S), n = 9; 10 μg/0.1 ml (EB10S), n = 9; 50 μg/0.1 ml (EB50S), n = 9 or 100 μg/0.1 ml (EB100S), n = 9) of EB 7 days post-ovariectomy. The uterine weights increased in line with the EB dose administered (F(5, 46) = 20.78, *p* < 0.0001). Superscript letters indicate statistical significance; *ps* < 0.05–0.0001.
(DOCX)

S1 Fig The relationship between freezing duration and uterine weight. Scatter plot of the cumulative freezing duration during the test trials versus the uterine weights of the estradiol-treated female mice with the regression lines superimposed. The filled circles represent the data points of each subject in all groups included in Experiment 2. The dashed line represents the estimated regression line (Y = 0.168X + 6.761, R^2^ = 0.104, F(1,32) = 3.73, *p* = 0.062) determined by a linear model, while the solid line represents the estimated regression line (Y = 0.004X^2^–0.831X + 37.710, R^2^ = 0.213, F(2,31) = 4.19, * *p* < 0.05) determined by a quadratic model.
(TIF)
