# Supplementary material for: The enhancement effect of estradiol on contextual fear conditioning in female mice
Source: PLoS One. 2018 May 15;13(5):e0197441. doi: 10.1371/journal.pone.0197441 (PMC5953469; doi:10.1371/journal.pone.0197441)
Supplement: S1 Table — Animals received a single subcutaneous (s.c.) injection of 0.1 ml of either 0.5 μg (EB0.5S, n = 9), 5 μg (EB5S, n = 7), or 50 μg (EB50S, n = 8) EB in oil, or oil vehicle alone (EB0S, n = 8). (DOCX) [file pone.0197441.s001.docx]

**S1 Table Behaviors recorded during the conditioning test in Experiment 1**

|  | **EB0S** | **EB0.5S** | **EB5S** | **EB50S** |
| --- | --- | --- | --- | --- |
| Stretching  (s) | 27.4 ± 5.6 | 27.2 ± 10.9 | 20.9 ± 6.4 | 45.9 ± 18.5 |
| Tail writhing  (s) | 22.7 ± 9.0 | 16.4 ± 8.4 | 21.8 ± 7.3 | 10.0 ± 6.2 |
| Rearing  (s) | 66.3 ± 11.5 | 71.2 ± 14.6 | 81.8 ± 12.9 | 54.8 ± 10.6 |
| Grooming  (s) | 13.6 ± 3.2 | 12.3 ± 2.5 | 11.6 ± 2.9 | 18.5 ± 5.8 |
