# Supplementary material for: The enhancement effect of estradiol on contextual fear conditioning in female mice
Source: PLoS One. 2018 May 15;13(5):e0197441. doi: 10.1371/journal.pone.0197441 (PMC5953469; doi:10.1371/journal.pone.0197441)
Supplement: S2 Table — Animals implanted s.c. with a Silastic capsule containing either 0.05 μg/0.1 ml (EB0.05L, n = 8), 0.5 μg/0.1 ml (EB0.5L, n = 8), 5 μg/0.1 ml (EB5L, n = 8), or 50 μg/0.1 ml (EB50L, n = 8) or an oil vehicle (EB0L, n = 8). Superscript letters indicate statistical significance; p<0.05. (DOCX) [file pone.0197441.s002.docx]

**S2 Table Behaviors recorded during the conditioning test in Experiment 2**

|  | **EB0L** | **EB0.05L** | **EB0.5L** | **EB5L** | **EB50L** |
| --- | --- | --- | --- | --- | --- |
| Stretching  (s) | 17.4 ± 4.2 | 18.0 ± 5.0 | 27.9 ± 4.7 | 27.5 ± 10.1 | 31.5 ± 6.7 |
| Tail tremor  (s) | 3.4 ± 1.2^a^ | 9.6 ± 2.7 | 5.6 ± 2.8 | 19.0 ± 5.4^b^ | 11.1 ± 3.9 |
| Rearing  (s) | 57.7 ± 9.8 | 54.3 ± 17.0 | 45.8 ± 8.7 | 42.5 ± 9.4 | 28.2 ± 10.5 |
| Grooming  (s) | 17.5 ± 1.9 | 13.2 ± 3.3 | 14.8 ± 1.8 | 10.2 ± 2.4 | 9.9 ± 2.5 |
