# Supplementary material for: The enhancement effect of estradiol on contextual fear conditioning in female mice
Source: PLoS One. 2018 May 15;13(5):e0197441. doi: 10.1371/journal.pone.0197441 (PMC5953469; doi:10.1371/journal.pone.0197441)
Supplement: S3 Table — Uterine weights were measured 2 days after a single administration of EB in a second group of animals. Animals received an s.c. injection of either oil vehicle (control, 0.1 ml sesame oil (EB0S), n = 8) or various doses (1 μg/0.1 ml (EB1S), n = 8; 5 μg/0.1 ml (EB5S), n = 9; 10 μg/0.1 ml (EB10S), n = 9; 50 μg/0.1 ml (EB50S), n = 9 or 100 μg/0.1 ml (EB100S), n = 9) of EB 7 days post-ovariectomy. The uterine weights increased in line with the EB dose administered (F(5, 46) = 20.78, p < 0.0001). Superscript letters indicate statistical significance; ps < 0.05–0.0001. (DOCX) [file pone.0197441.s003.docx]

**S3 Table Uterine weight** **2 days after s.c. administration of EB**

| EB dose | EB0S | EB1S | EB5S | EB10S | EB50S | EB100S |
| --- | --- | --- | --- | --- | --- | --- |
| Uterine weight (mg) | 35.8 ± 8.1^a^ | 55.8 ± 5.9^ab^ | 68.3 ± 3.8^bc^ | 86.6 ± 7.4^cd^ | 120.2 ± 7.6^e^ | 113.4 ± 8.9^de^ |
