# Supplementary figures and images for: The enhancement effect of estradiol on contextual fear conditioning in female mice
Source: PLoS One. 2018 May 15;13(5):e0197441. doi: 10.1371/journal.pone.0197441 (PMC5953469; doi:10.1371/journal.pone.0197441)

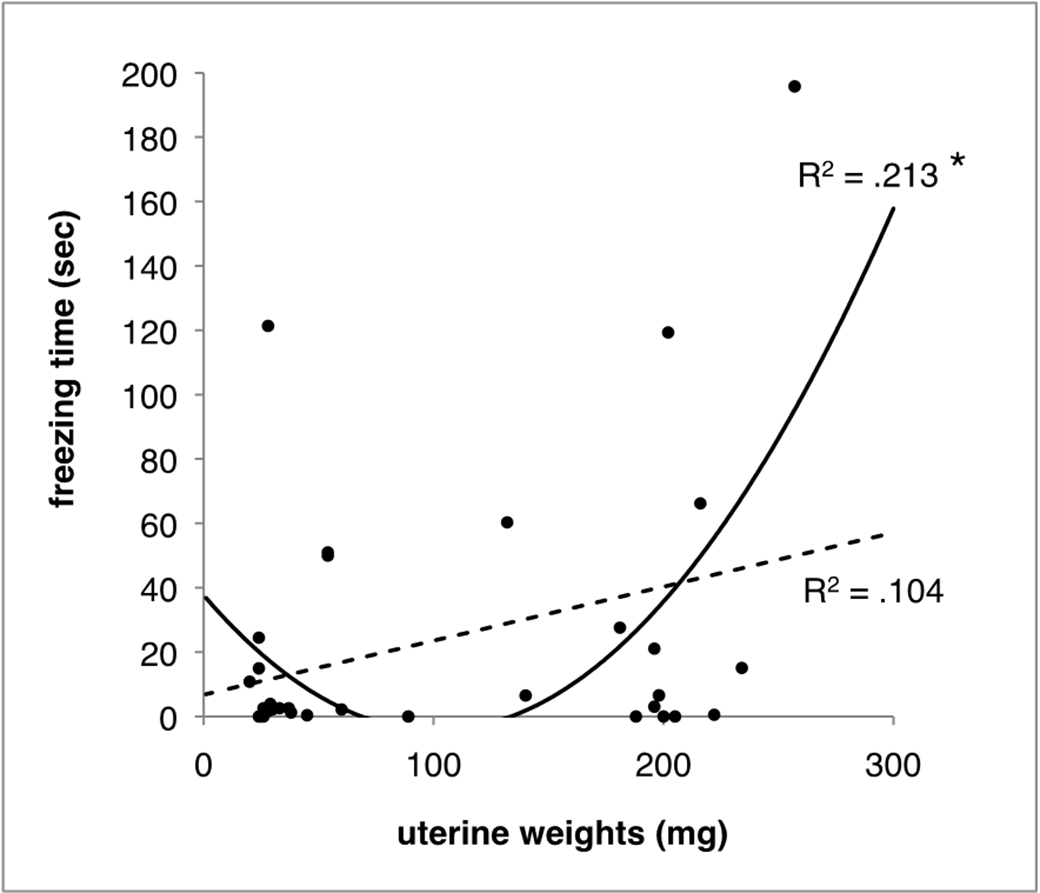

Supplement: S1 Fig — Scatter plot of the cumulative freezing duration during the test trials versus the uterine weights of the estradiol-treated female mice with the regression lines superimposed. The filled circles represent the data points of each subject in all groups included in Experiment 2. The dashed line represents the estimated regression line (Y = 0.168X + 6.761, R2 = 0.104, F(1,32) = 3.73, p = 0.062) determined by a linear model, while the solid line represents the estimated regression line (Y = 0.004X2–0.831X + 37.710, R2 = 0.213, F(2,31) = 4.19, * p < 0.05) determined by a quadratic model. (TIF) [file pone.0197441.s004.tif]
